# Trait self-awareness predicts perceptions of choice meaningfulness in a decision-making task

**DOI:** 10.1186/s13104-018-3191-2

**Published:** 2018-01-25

**Authors:** Noam Dishon, Julian A. Oldmeadow, Jordy Kaufman

**Affiliations:** Department of Psychological Sciences, Faculty of Health, Arts and Design, Swinburne University of Technology, Hawthorn, Australia

**Keywords:** Trait self-awareness, Choice meaningfulness, Decision-making

## Abstract

**Objective:**

Seminal theorists such as Erikson, Bruner, Frankl and Rogers have underscored the importance of meaning in psychological life. However contemporary scholars interested in meaning have noted that further investigation of the individual differences associated with meaning-making is still needed. In the present study we explored whether individual differences in trait self-awareness were associated with perceptions of choice meaningfulness in a decision-making task.

**Results:**

All participants engaged in a decision-making task wherein they were asked to choose their preferred painting out of pairs of sequentially presented abstract paintings. Participants in the experimental condition were provided with feedback that their choices had been diagnostic of important personality characteristics whereas those in the control condition were not. All participants were then prompted to reflect on their choices before rating the subjective meaningfulness that they associated with their choices and completing measures to assess trait self-awareness. As anticipated, persons with higher levels of trait self-awareness tended to seek out and find more meaning compared to those lower in trait self-awareness. However contrary to expectations, feedback about the self-relevance of choices did not moderate perceptions of choice meaningfulness. Implications of these findings as well as directions for future research are also discussed.

## Introduction

Several notable humanistic, existential, and developmental scholars have theorised that meaning-making plays a significant role in psychological life [1–3]. However, developing a richer understanding of the individual differences that are predictive of meaning-making tendencies remains important [4]. To help address this issue, in the present study we investigate whether individual differences in trait self-awareness predict meaning-making within a decision-making context.

### Meaning

Meaning-making describes the process wherein one imbues a particular event or phenomenon with a sense of personal significance whereas subjective meaningfulness reflects the experience of *feeling* as though something matters [5]. Whilst researchers interested in meaning-making and meaningfulness have often explored these issues as they relate to significant life events [e.g. 6] the idea that they should also intersect with day-to-day life is a point that has been made by both Frankl [2] and Maddi [7]. For example, Maddi writes that “…personal meaning derives from the individual decisions people make every day” (p. 57) and Frankl articulates that humans possess a ‘will to meaning’ which is continuously expressed in the day-to-day activities of our lives. Several studies have provided empirical support for the idea that meaning matters in the context of day-to-day life [8–10]. Other researchers have extended this idea further by demonstrating that perceptions of meaningfulness for everyday actions can be manipulated by exposure to primes [11].

### Self-awareness

Within the literature self-awareness has been conceptualized as possessing both state like and trait like qualities. For example, self-awareness has been described both as the experience of “becoming the object of one’s own attention” (p. 808) [12] which reflects a state like experience, and it has also been conceptualized as an individual’s capacity to identify, process and store self-related information, which is more reflective of a persistent trait like characteristic [12]. The idea that self-awareness has both state like and trait like qualities also connects with the mindfulness and borderline personality literatures. For example, mindfulness, which is a state in which one purposefully reflects inwardly on their self-experience, appears to reflect state self-awareness [13]. Alternatively, borderline personality disorder (BPD) is a disorder characterized by an unstable sense of self and marked impairment in self-understanding and self-knowledge. The fact that BPD is conceptualized as a personality (and not mood) disorder reflects the idea that this impairment in self-awareness is persistent and trait like rather than transient and state like [14].

### The present study

In the present study we investigated whether individual differences in trait self-awareness (which we defined as the degree to which one possesses knowledge, insight and understanding of their internal self-related experiences) in a non-clinical population could predict meaning-making with a decision-making context. We hypothesized the existence of a relationship between trait self-awareness and meaning-making and used a decision-making event for several reasons. First, research has demonstrated that persons affected by BPD (a disorder characterized by persistent deficits in self-awareness) tend to find meaning-making more difficult compared to healthy controls [15] and given that trait theories of personality often conceptualize personality characteristics as existing on a continuum, it stands to reason that even in non-clinical populations, as trait self-awareness decreases so too might tendencies to engage in meaning-making. Second, as noted previously it has been suggested by Maddi [7] that meaning-making and everyday decision-making share a close relationship with one another. And third, simple decision-making exercises are conducive to both experimental and diagnostic testing [16]. Additionally, because previous research has demonstrated that exposure to external stimuli can impact perceptions of meaningfulness [11], in the present study, we also sought to explore the strength of the possible relationship between trait self-awareness and perceptions of meaningfulness by investigating if such a relationship was moderated by (or robust to) the influence of external feedback about choice self-relevance and meaningfulness.

### Hypotheses

H1: It was expected that participants higher in trait self-awareness would perceive their choices as being more subjectively meaningful than those lower in trait self-awareness.
H2: It was predicted that participants provided with feedback that their choices were diagnostic of important personality characteristics and therefore self-relevant and meaningful would perceive their choices as more subjectively meaningful than participants who were not provided with this feedback.

## Main text

### Methods

#### Participants

Participants were 177 undergraduate psychology students (141 female, 31 male, five unidentified) from an Australian university aged 18–57 years (*M* = 31.42, *SD* = 10.09) who volunteered to participate in the study for course credit.

### Materials

#### Trait self-awareness

Because we conceptualized trait self-awareness as a combination of self-insight, self-understanding and self-knowledge, we measured trait self-awareness as a function of participant scores on the Self-Reflection and Insight Scale^1^ [17] (Cronbach’s α = .87) and Sense of Self Scale [18] (Cronbach’s α = .88). Previous research has replicated the original factor structures of both measures and demonstrated that both scales possess strong internal consistency [e.g. 19, 20]. Items were scored so that higher scores represented greater levels of self-reflection and insight and stronger sense of self. Correlational analysis demonstrated the two measures were positively and significantly associated (*r* = .32, *p* < .001). To reflect an overall trait self-awareness score both measures were then summed with higher scores indicating greater levels of trait self-awareness (Cronbach’s α = .88).

#### Subjective meaningfulness

For the purposes of the present study we developed four items to measure perceptions of subjective meaningfulness for painting choices (i.e. “*My choices were meaningless*”, “*My choices were genuine*”, “*My choices were random*”, “*If I were to choose again*, *I would choose the same paintings*”). Participants indicated their level of agreement with each item on a 5-point scale. Responses were coded so that higher scores reflected a greater perception of choice meaningfulness. Principal axis factoring with promax rotation found all items loaded on a single factor which accounted for 43.36% of the variance (all factor loadings ≥ .60). Responses were then averaged to calculate an overall subjective meaningfulness score (Cronbach’s α = .75).

#### Stimuli

Six artworks by Wassily Kandisky and six artworks by Paul Klee were sequentially presented in mixed pairs to participants as the painting stimuli. These artists’ works were chosen because they are abstract in nature such that any meaning attributed to them is highly subjective, and also because they have been successfully used in previous research utilizing choice behavior [e.g. 21].

### Procedure

Participants completed the experiment online. After consenting to participate, participants were sequentially presented with six pairs of paintings and asked to choose a preferred painting out of each pair. Participants where then randomly assigned to either the *self*-*relevant*/*meaningful* or the *self*-*irrelevant*/*meaningless* condition. Those assigned to the self-relevant/meaningful condition where presented with the text below:

“In this study we are interested in how much insight people have into their choices, and whether this varies across different types of choice situations. In the previous task, you were asked to choose between pairs of paintings. These choices were in fact extremely meaningful. Previous research has demonstrated that people’s preferences between those particular pairs of paintings reveals a great deal about their personality. Your choices corresponded to a range of deep and stable aspects of your personality. In the next section, we would like to ask you a few questions about your choices.”

Alternatively, participants assigned to the self-irrelevant/meaningless condition were presented with the following text:

“In this study we are interested in how much insight people have into their choices, and whether this varies across different types of choice situations. In the previous task, you were asked to choose between similar paintings. Although these choices were meaningless we are nevertheless interested in how you made your selections. In the next section, we would like to ask you a few questions about your choices.”

Participants were then prompted to self-reflect on their choices. To do so, participants were asked to reflect on and write about the reasons behind their choices in an open text box. To further encourage self-reflection, a list of 15 potential reasons for choice selections was then presented on screen and participants were asked to reflect on an indicate the extent to which each reason applied to them. Participants were then presented with the measure of subjective meaningfulness, individual difference questionnaires and demographic questions (age and gender).

### Results

#### Effect of the experimental manipulation on trait self-awareness

An independent samples t-test revealed that there was no significant difference (*p* = .669) in trait self-awareness scores between participants in the self-relevant condition (*M* = 7.25, *SD* = 1.10, n = 89) and in the self-irrelevant condition (*M* = 7.19, *SD* = .97, n = 88). This demonstrated that the IV of trait self-awareness was not impacted by the experimental manipulation.

#### Correlational analysis

A correlational analysis examined whether a linear relationship existed between trait self-awareness and perceptions of choice meaningfulness. A significant positive linear relationship between trait self-awareness and perceptions of choice meaningfulness was found in both the self-relevant condition (*r* = .34, *p* = .001) and the self-irrelevant condition (*r* = .29, *p* = .005).

#### Moderated regression analysis

To investigate whether the feedback condition moderated the relationship between trait self-awareness and perceptions of choice meaningfulness a moderated regression was conducted to assess the increase in variance accounted for with the addition of an interaction term between trait self-awareness and feedback condition (Stage 2) on a main effects model (Stage 1).^2^ Multicollinearity was ruled out as all tolerance values were greater than .43. The model was significant at Stage 1 *R*^2^ = .102, *F*(2,174) = 9.847, *p* < .001. At Stage 1 a significant main effect of trait self-awareness was found (***β*** = .187, *SE* = .042, *p* < .001) however the main effect of condition was not significant (*p* = .592). At Stage 2, the main effect of trait self-awareness remained significant (***β*** = .190, *SE* = .065, *p* = .004) and the main effect of condition remained non-significant (*p* = .593). Additionally at Stage 2 the addition of the interaction term revealed that self-relevance feedback did not moderate the effect of trait self-awareness on meaningfulness scores as it was not significant *F*(1,173) = .004, *p* = .952.

### Discussion

The present study investigated whether individual differences in trait self-awareness predicted perceptions of subjective meaningfulness for choices in a decision-making task. We also investigated the possibility that feedback relating to choice self-relevance and meaningfulness could moderate perceptions of choice meaningfulness. As anticipated, participants higher in trait self-awareness tended to perceive their choices as more subjectively meaningful than those lower in trait self-awareness irrespective of condition. However contrary to expectations, the relationship between trait self-awareness and choice meaningfulness was not moderated by feedback condition and participants did not perceive their choices to be any more meaningful when they were provided with feedback that their choices had been self-relevant and personally meaningful.

### Implications

BPD is a condition which is characterized by reduced self-awareness and previous research has demonstrated that individuals affected by BPD find meaning-making more challenging than healthy controls [e.g. 15]. The results of this study extend that research by demonstrating that even in a non-clinical sample, persons higher in trait self-awareness are more likely to seek out and find meaning compared to those lower in trait self-awareness.

### Conclusion

Numerous influential theorists within psychology have suggested that meaning-making plays a central role in psychological life. In the present study we investigated if individual differences in trait self-awareness predicted perceptions of choice meaningfulness in a decision-making context. Results demonstrated that within a choice context, individuals with higher levels of trait self-awareness are more likely to seek out and find meaning compared to those lower in trait self-awareness and that this effect is robust to external feedback about choice self-relevance.

## Limitations

In the present study we measured trait self-awareness by combining existing measures of self-reflection and insight, and sense of self. Whilst there was strong theoretical ground for this, in future the development of a dedicated trait self-awareness measure could prove beneficial. Additionally, it remains unclear whether participants higher in trait self-awareness perceived their choices as more subjectively meaningful because of a dispositional pull towards engaging in explicit self-reflection and meaning-making or, because they have a natural tendency to imbue experiences with greater levels of meaning via automatic or implicit processes. To explore this question an experimental design that involved manipulating the presence or absence of self-reflection would be worthwhile.

## Abbreviation

BPD: borderline personality disorder

## Footnotes

1 One of the original 20 items was omitted from the analysis due a presentation error.

2 Trait self-awareness scores were mean centered prior to the moderated regression analysis.
